# Abnormalities in gray matter microstructure in young adults with 22q11.2 deletion syndrome

**DOI:** 10.1016/j.nicl.2018.101611

**Published:** 2018-11-27

**Authors:** Zora Kikinis, Nikos Makris, Valerie J. Sydnor, Sylvain Bouix, Ofer Pasternak, Ioana L. Coman, Kevin M. Antshel, Wanda Fremont, Marek R. Kubicki, Martha E. Shenton, Wendy R. Kates, Yogesh Rathi

**Affiliations:** a Department of Psychiatry, Psychiatry Neuroimaging Laboratory, Brigham and Women's Hospital, Harvard Medical School, Zora Kikinis, 1249 Boylston Street, Boston, MA 02215, USA; b Departments of Psychiatry and Neurology, Massachusetts General Hospital, Harvard Medical School, Boston, MA, USA; c Department of Radiology, Brigham and Women's Hospital, Harvard Medical School, Boston, MA, USA; d Department of Psychiatry, SUNY Upstate Medical University, Syracuse, NY, USA; e Department of Computer Science, SUNY Oswego, Oswego, NY, USA; f Department of Psychology, Syracuse University, Syracuse, NY, USA; g VA Boston Healthcare System, Brockton, MA, USA

**Keywords:** 22q11.2 deletion syndrome, Gray matter, Diffusion magnetic resonance imaging, Fractional anisotropy, Response inhibition, Cognition

## Abstract

**Background:**

22q11.2 Deletion Syndrome (22q11DS) is a genetic, neurodevelopmental disorder characterized by a chromosomal deletion and a distinct cognitive profile. Although abnormalities in the macrostructure of the cortex have been identified in individuals with 22q11DS, it is not known if there are additional microstructural changes in gray matter regions in this syndrome, and/or if such microstructural changes are associated with cognitive functioning.

**Methods:**

This study employed a novel diffusion MRI measure, the Heterogeneity of Fractional Anisotropy (HFA), to examine variability in the microstructural organization of the cortex in healthy young adults (*N* = 30) and those with 22q11DS (*N* = 56). Diffusion MRI, structural MRI, clinical and cognitive data were acquired.

**Results:**

Compared to controls, individuals with 22q11DS evinced increased HFA in cortical association (*p* = .003, *d* = 0.86) and paralimbic (*p* < .0001, *d* = 1.2) brain areas, whereas no significant differences were found between the two groups in primary cortical brain areas. Additionally, increased HFA of the right paralimbic area was associated with poorer performance on tests of response inhibition, *i.e.*, the Stroop Test (*rho* = −0.37 *p* = .005) and the Gordon Diagnostic System Vigilance Commission (*rho* = −0.41 *p* = .002) in the 22q11DS group. No significant correlations were found between HFA and cognitive abilities in the healthy control group.

**Conclusions:**

These findings suggest that cortical microstructural disorganization may be a neural correlate of response inhibition in individuals with 22q11DS. Given that the migration pattern of neural crest cells is disrupted at the time of early brain development in 22q11DS, we hypothesize that these neural alterations may be neurodevelopmental in origin, and reflect cortical dysfunction associated with cognitive deficits.

## Highlights

• Microstructure of brain gray matter can be analyzed now *in vivo* using diffusion MRI.
• Abnormalities in microstructure are present in people with 22q11 Deletion Syndrome.
• Changes in microstructure were associated with subject's executive functioning.

## 1 Introduction

Abnormalities in gray matter in the brain are present in individuals with 22q11.2 deletion syndrome (22q11DS). More specifically, studies using structural MRI have reported macrostructural changes, such as changes in volume, surface area, and cortical thickness of gray matter regions in 22q11DS (Ramanathan et al., 2016; Bakker et al., 2016; Schmitt et al., 2016; Jalbrzikowski et al., 2013; Schmitt et al., 2015; Schaer et al., 2009; Kates et al., 2006; Berhanu et al., 2017), but the microstructure of gray matter tissue has yet to be studied *in vivo*. Newly developed diffusion MRI (dMRI) techniques, however, now make it possible to investigate microstructural architectural abnormalities in gray matter. While dMRI has typically been used to investigate the microstructure of white matter, it has more recently been successfully employed to explore changes in gray matter architecture.

The most widely studied dMRI output measure is fractional anisotropy (FA). FA is believed to detect microstructural changes, such as changes in axonal myelination in white matter, whereas in gray matter, FA might be less of a measure of myeloarchitecture, but rather a measure of cytoarchitecture (Assaf, 2018). The vast majority of studies examine average FA values within a given region of interest, and compare FA averages between groups. This averaged measure, however, does not convey all of the available information about a given region. For example, two individuals could have the same average FA value in a given region of interest, but evince significantly different variance in FA measures. Consequently, the HFA measure was proposed, which calculated the heterogeneity of *FA* values in *gray matter.*

In the first study to examine gray matter heterogeneity, HFA was observed to increase across the life span of healthy individuals in primary and association areas of the brain (Rathi et al., 2014), but not in paralimbic areas. These findings are in line with evidence of a decline in motor and cognitive abilities, but preserved emotional regulation in elderly people (Leclerc and Kensinger, 2008; Fling et al., 2012; Riecker et al., 2006). This finding, which was interpreted as representing age-related decreased structural integrity and increased degeneration in primary and association areas, provides initial evidence that tissue heterogeneity in specific cortical regions may be associated with behavior, cognition, and emotional traits. Furthermore, this finding suggests that increased HFA may be associated with a more pathological state of brain tissue. Based on findings from this study, we aimed to explore whether individuals with neurodevelopmental abnormalities, such as those with 22q11DS, would exhibit greater variability (heterogeneity) in gray matter microstructure.

22q11DS is a genetic disorder characterized by a deletion of over 50 genes on one copy of chromosome 22. Deviant neurodevelopment in individuals with this syndrome is evinced by subtle structural malformation of the face, thymus gland, parathyroid gland, and branchial arch arteries. The pathogenesis of this disorder involves the disruption of neural crest cells during early development, a theory that has been corroborated by animal studies (Scambler, 2000; Walker and Trainor, 2006). Further, disturbances of cortical gray matter are revealed in postmortem studies in individuals with 22q11DS (Kiehl et al., 2009). In addition, in a mouse model of 22q11DS, development of the cortical layer 2/3 is disrupted, such as the number and the placement of projection neurons and interneurons are abnormal (Meechan et al., 2015). Such alterations in cortical circuits, have been associated with cognitive ability (Meechan et al., 2009; Meechan et al., 2011), suggesting a possible connection between gray matter and cognitive functioning in 22q11DS. Consistent with the aforementioned findings, impairments in cognitive functioning occur in many children with 22q11DS. Children with this syndrome experience mild to moderate intellectual disability with an average FSIQ of 75 (Moss et al., 1999; Niklasson et al., 2009; Niklasson and Gillberg, 2010; Niklasson et al. 2002; Walker et al., 2006) and difficulties in a range of cognitive abilities, including cognitive control (Bish et al., 2005; Sobin et al., 2005). This wide range of deficits among individuals with 22q11DS allows for the exploration of neuroanatomical substrates that might be associated with cognitive function.

**Fig. 1:**
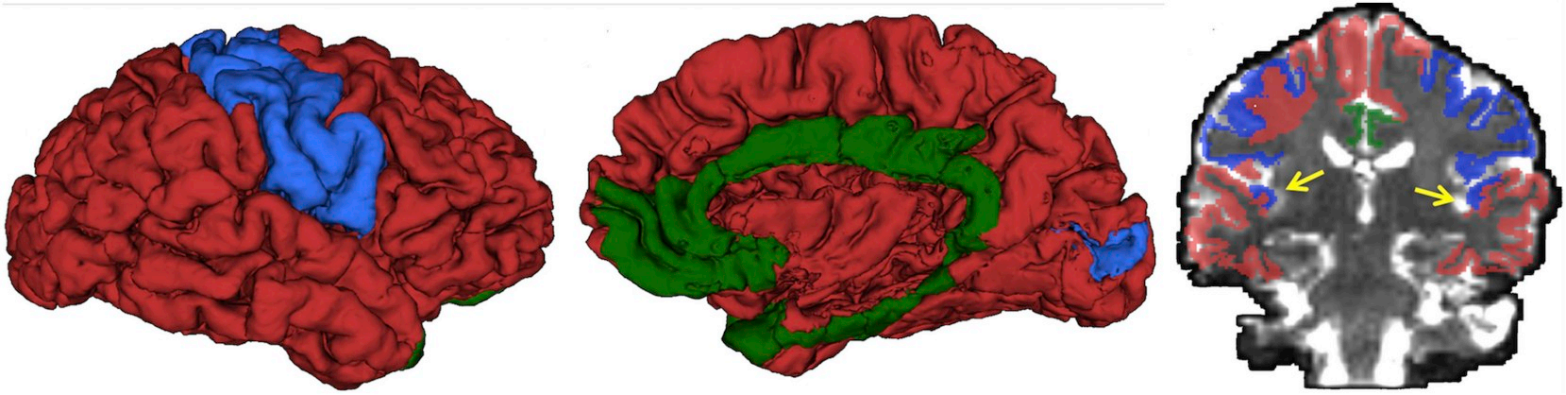
Cortical functional areas, primary (blue), associative (red), and paralimbic (green), are presented in lateral view (left), medial view (center) and coronal view (right). The arrow in the panel on the right points to primary auditory areas. The left and right primary auditory areas are located in the temporal lobes and colored in blue. (For interpretation of the references to color in this figure legend, the reader is referred to the web version of this article.)

Cognitive abilities rely on the integrated functioning of diverse brain regions. As proposed by Mesulam, the cortex can be divided based on functionality into three major functional regions, namely primary, associative, and paralimbic areas (Fig. 1) (Mesulam, 2000). This parcellation of functional regions in the brain is further based on the knowledge that processing of sensory information begins in primary areas, and continues in nearby associative areas, which are responsible for unimodal and multimodal perceptual processing. Paralimbic areas integrate sensory information with cognitive, emotional, and visceral information. This integration of information across associative and paralimbic areas is required for high level cognitive processing (Mesulam, 2000). Deficits in cognitive abilities, such as those observed in individuals with 22q11DS, may therefore be the result of pathology in associative and paralimbic areas that process higher order information.

The aim of the present study was to investigate the structural architecture of gray matter, and its association with cognitive functioning, in young adults with 22q11DS and in healthy controls. We explored the microstructure of gray matter using dMRI data by calculating average FA and heterogeneity of FA (HFA) in three brain regions of interest (primary, association, and paralimbic areas). We hypothesize that heterogeneity of FA will be increased in individuals with 22q11DS and that this increase will be associated negatively with cognitive abilities. Our approach to the study of gray matter in 22q11DS and healthy subjects may provide new information about gray matter tissue abnormalities that underlie pathology of the brain.

## 2 Materials and methods

### 2.1 Subjects

Demographic, clinical, neuropsychological, and MRI data were acquired from 56 participants with 22q11DS (mean age 20.9 years) and 30 age and gender matched controls (mean age 20.8 years) (Table 1). The control sample consisted of unaffected siblings (*N* = 11) and community controls (*N* = 19). Because sibling and community controls did not differ on demographic or any of the FA variables (all *p*-values > .05), we combined them into one control sample to increase statistical power. Upon enrollment in the study, 22q11.2 deletion syndrome was confirmed with fluorescence *in situ* hybridization (FISH). Among the subjects with 22q11DS, four individuals were diagnosed with a psychotic disorder, and six were prescribed antipsychotic medication. The diagnosis of psychosis is not surprising given that there is an increased incidence of psychosis in individuals with 22q11DS in adulthood (Schneider et al., 2014).

**Table 1:** Characteristics of study subjects.

	Healthy controls	22q11DS
	(N = 30)	(N = 56)
Age (in years; mean ± SD)	20.8 (1.5)	20.9 (2.2)
Age range (in years)	18.9–23.8	17.8–25.9
Gender (% females)	51%	50%
FSIQ (mean ± SD)	110 (16)⁎	75 (12)⁎
Range	72–142	52–103
Medication		
Anti-depressant/anti-anxiety	1	15
Stimulants	2	6
Mood stabilizers	0	4
Antipsychotic medications	0	6

22q11DS: 22q11Deletion Syndrome; FSIQ: full scale IQ.

⁎ *p* < .001.

The data were acquired as part of a longitudinal study to assess risk factors for psychosis development in 22q11DS, conducted at SUNY Upstate Medical University, Syracuse, NY. The data used in this report are from the 4th time point in this longitudinal study. DMRI data acquisitions of the 4th time point are of high quality to allow analysis of microstructure in the cortex. The following publications have also used data from this same time point in the study and focused on investigation of structural analysis of brain gray and white matter and connectivity using functional MRI: (Olszewski et al., 2017; Berhanu et al., 2017; Schreiner et al., 2017; Thompson et al., 2017; Hamsho et al., 2017; Antshel et al., 2016; Mattiaccio et al., 2016; McCarthy et al., 2015; Tylee et al., 2017; Mattiaccio et al., 2018; Taylor et al., 2018).

### 2.2 Image acquisition

Structural MRI and diffusion-weighted images (DWI) were acquired on a 3T Siemens Magnetom Tim Trio scanner (Siemens Medical Solutions, Erlangen, Germany). The high-resolution structural scan consisted of an ultrafast gradient echo 3D sequence (MPRAGE) with PAT k-space-based algorithm GRAPPA. The parameters were: echo time = 3.31 ms; repetition time = 2530 ms; matrix size = 256 × 256; field of view (FOV) = 256 mm; slice thickness = 1 mm. The DWI sequence acquired 64 transverse slices with no gaps and 2.0 mm nominal isotropic resolution (TR/TE = 8600/93 ms, FOV = 244 × 244, data matrix = 96 × 96, zero-filled and reconstructed to 256 × 256). Diffusion weighting was performed along 64 directions with a *b* value = 900 s/mm^2^. One non-diffusion weighted volume (b_0_) was acquired within each DWI dataset. A high resolution T2 scan was also obtained to allow for enhanced registration of structural MR images to DWIs.

### 2.3 Image processing

Structural and diffusion images were visually inspected for motion artifacts. DWIs were additionally inspected for signal dropouts. All images passed quality control procedures. An in-house script was used to correct for eddy current distortions and head motion by registering each diffusion-weighted volume to the b_0_ volume using the FMRIB software library (FSL) (http://fsl.fmrib.ox.ac.uk) Linear Images Registration Tool known as “FLIRT”.

**Fig. 2:**
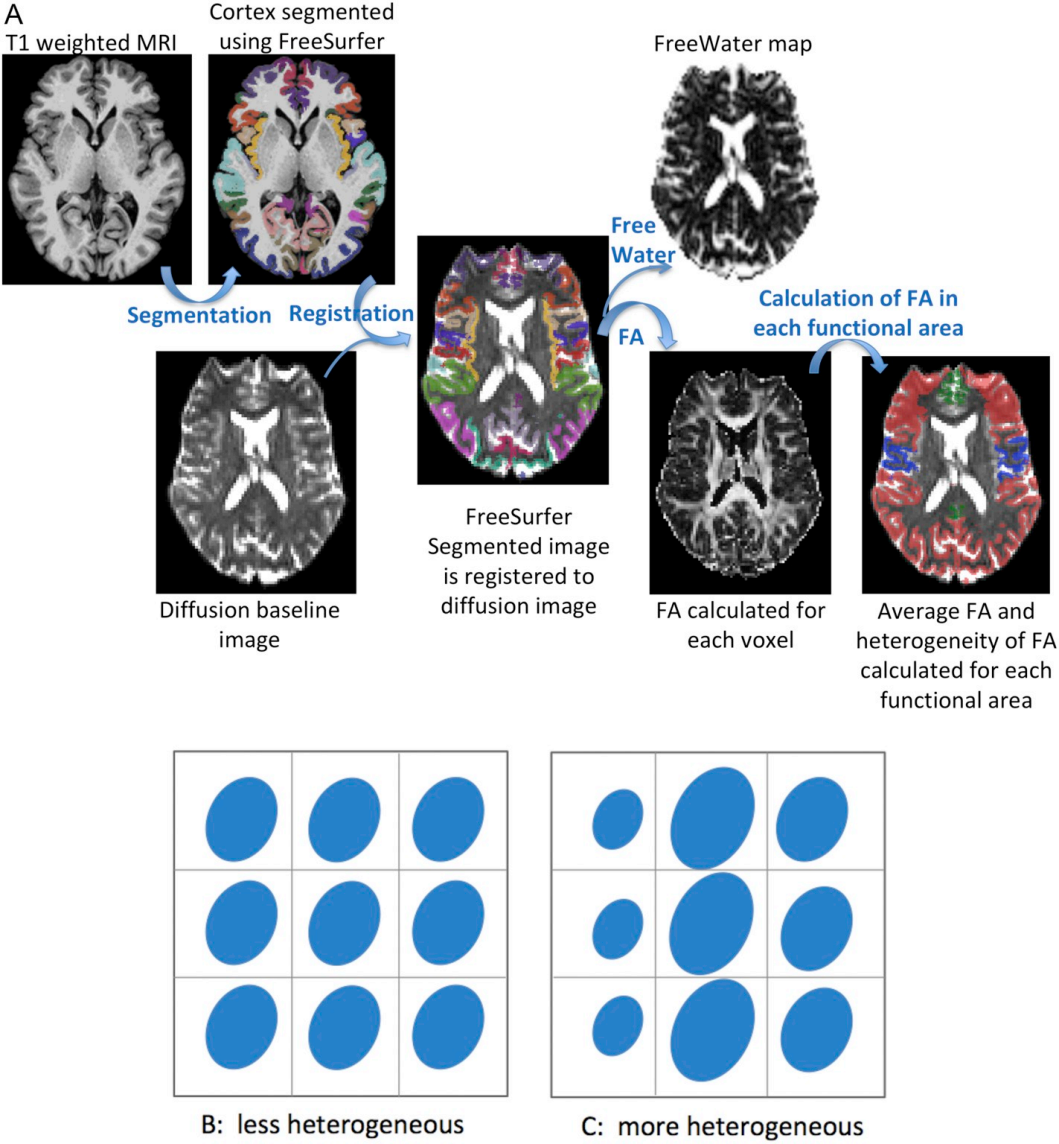
Panel A: Overview of image post-processing. Structural MRI image (T1 weighted) was segmented in an automated manner using the FreeSurfer software to parcellate the cortex. This segmented image was then registered to the diffusion image (using the diffusion baseline image). Fractional Anisotropy (FA) was calculated for each voxel. In the calculation of FA the compartment of free-water was removed from each voxel. Lastly, functional areas were assembled using FreeSurfer parcellations, and average FA and HFA were computed for primary, association, and paralimbic areas in the brain. Panel B and C: A scheme to illustrate a ‘less heterogeneous’ and ‘more heterogeneous’ distribution of FA (ellipsoids in blue) in a region of interest (of the size 3 × 3 voxels). While the average FA is the same in panel B and C, heterogeneity varies. (For interpretation of the references to color in this figure legend, the reader is referred to the web version of this article.)

Following the motion and distortion corrections, each structural MRI image (T1) was parcellated into cortical regions using FreeSurfer software (http://surfer.nmr.mgh.harvard.edu). The FreeSurfer parcellations were then registered to DWIs by performing a rigid registration from the T1 to the T2 image, followed by a non-rigid registration from the T2 to the b_0_ image using ANTs (Avants et al., 2014). The motion and eddy corrected DWIs were used to calculate FA at each voxel. In the calculation of FA, free-water correction was applied (Pasternak et al., 2009) to prevent partial volume with CSF, which is expected especially on the boundary of gray matter and CSF. Fig. 2A summarizes the post-processing image pipeline.

Average FA was calculated for each region of interest. Cortical regions of interest, namely primary, paralimbic, and association areas, were assembled using FreeSurfer parcellations (see below), and average FA and heterogeneity of FA (HFA) values were obtained for each of the three cortical areas in each hemisphere, in each subject. The heterogeneity in FA is mathematically defined as follows:

$$\mathrm{HFA}=\frac{1}{N^{2}}\sum_{i=1}^{N}\sum_{j=1}^{N}{\left|m_{i}-m_{j}\right|}^{2}\;$$

where N stands for the number of voxels in the region of interest (primary, association or paralimbic areas), and m_i_ is the FA value at each voxel. In the calculation of HFA, all FA values that were outside of three standard deviations from the mean were excluded to prevent the inclusion of outlier values, such as those affected by partial volume effects with CSF. In summary, the heterogeneity measure, HFA, depicts the statistical variance of FA within a region of interest (Fig. 2B) (Rathi et al., 2014).

### 2.4 Assembly of primary, associative, paralimbic areas

FreeSurfer parcellations were used to construct the primary, associative and paralimbic areas (Fig. 1). The primary area included the following FreeSurfer parcellated cortical regions: postcentral, precentral, pericalcerine, and transverse temporal. The paralimbic area included the caudal anterior cingulate, isthmus cingulate, posterior cingulate, rostral anterior cingulate, parahippocampal, entorhinal, temporal pole, and medial orbito-frontal. The associative areas included the remaining 22 cortical regions.

### 2.5 Neuropsychological measures

Neuropsychological and clinical tests were used to assess cognitive abilities. Associative areas are thought to subserve skills that are assessed with the Wisconsin Card Sorting Test (WCST) (Heaton et al. 1993), the Wechsler Adult Intelligence Scale Working Memory Index III (WAIS WMI) (Wechsler, 1997), and the California Verbal Learning Test (CVLT) (Woods et al., 2006). The paralimbic areas subserve skills assessed by the Stroop task (Stroop, 1935), the Gordon Diagnostic system (GDS) Distractibility Commission, the GDS Vigilance Commission and the Gordon CPT Vigilance Omission tests (Gordon, 1988). Neuropsychological and clinical tests were administered on the same day as imaging data.

### 2.6 Statistical analysis

MANOVA was used to compare the effect of diagnosis (control subjects *versus* 22q11DS), hemisphere (left *versus* right), and brain region (primary, association, paralimbic areas) on FA. Similarly, a second MANOVA was performed for HFA to assess the effect of diagnosis, hemisphere, and region, and was followed by independent two-sample *t*-tests using a two-tailed significance level. The probability of null hypothesis p (*p*-value) being rejected was corrected for multiple comparisons using Bonferroni correction. Effect sizes and Cohen's *d* were also calculated (Cohen, 1988). The degree of linear correlation between HFA and performance on neuropsychological tests was assessed using Spearman's correlation coefficient *rho* (the scores on neuropsychological tests were not distributed normally); correlation between HFA and cortical thickness was assessed using Pearson's correlation coefficient *r* (values of both variables were normally distributed)*.* The normal distribution of the data was tested using the Shapiro-Wilk test. Statistical analyses were performed in IBM-SPSS software, version 24.

## 3 Results

### 3.1 Average FA and heterogeneity in FA in distinct functional brain regions

To examine the structural organization of the cortex in individuals with 22q11DS and in healthy control subjects, we computed average FA and heterogeneity in FA in the three main functional zones. We employed both measures as each provides unique information about cortical microstructure.

**Fig. 3:**
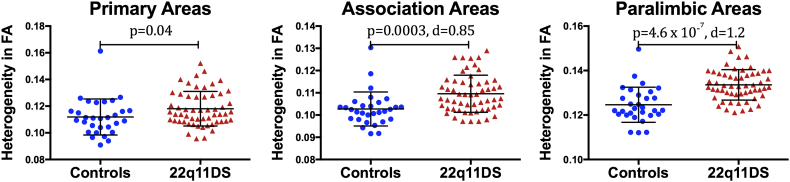
Heterogeneity in FA (HFA) in primary, association, and paralimbic areas was compared between healthy controls (HC) and individuals with 22q11 Deletion Syndrome (22q11DS). The values for HC are represented as blue circles and those for subjects with 22q11DS are presented as red triangles. The error bars represent the mean and the standard deviation (SD) of the t-test. The probability value *p* and the effect size *d* is given. Statistically significant differences at *p* < .016 (two-tailed, Bonferroni corrected). (For interpretation of the references to color in this figure legend, the reader is referred to the web version of this article.)

We performed MANOVAs to assess the effect of diagnosis, hemisphere, and functional zone on average FA and HFA. There was no effect of diagnosis on average FA (Table 2). However, for HFA, there was an effect of diagnosis (control *versus* 22q11DS group) and brain region (primary, association, paralimbic areas), but no effect of hemisphere (left, right) (Table 2). We followed up with independent-two-sample *t*-tests to determine if there were differences in HFA between the two groups (controls and 22q11DS) in each of the three brain areas. Because the MANOVA did not indicate any effect of hemisphere, we averaged the values of HFA for the left and right hemisphere for the t-test analyses. This resulted in three t-tests (Table 3, Fig. 3). To reduce the chance of obtaining false-positive results (type I errors), a Bonferroni correction was used with a statistical significance set at *p* ≤ .016. We found no statistically significant differences in HFA for primary areas (*p* > .016). However, individuals with 22q11DS showed a statistically significantly increase in HFA in association (*p* = .003) and in paralimbic areas (*p* < .0001) when compared to controls (Table 3, Fig. 3). In summary, these results demonstrate increased variability in the organization of gray matter in association and paralimbic areas in 22q11DS, but not in primary areas of the brain.

**Table 2:** MANOVA for average fractional anisotropy (FA) and heterogeneity in FA (HFA).

DTI measure	source	df	F	Sig. p	Partial Eta squared
FAt	Main effect of diagnosis	1	2.9	0.095	0.03
	Main effect of hemisphere	1	4.1	0.046	0.05
	Main effect of region	1.6	488.0	**<0.0001**	0.85
HFAT	Main effect of diagnosis	1	16.2	**<0.0001**	0.16
	Main effect of hemisphere	1	2.6	0.108	0.03
	Main effect of region	1.3	214.4	**<0.0001**	0.72

Probability values *p* are given in bold face when significant after correction for multiple comparison.

**Table 3:** Independent two-sample t-tests for Heterogeneity in FA (HFA) between the 22q11DS and control group.

Heterogeneity in FA	t	df	p	Mean difference	95% confidence interval		Effect size d
HFA primary area	2.1	84	0.0406645	−0.0062	−0.0121	−0.0003	0.47
HFA association area	3.7	84	**0.0003447**	−0.0069	−0.0105	−0.0032	**0.85**
HFA paralimbic area	5.5	84	**0.0000005**	−0.0090	−0.0122	−0.0057	**1.21**

Probability values *p* are given in bold face when significant after correction for multiple comparison.

### 3.2 Partial-volume effect

Although diffusion MRIs were acquired with a spatial resolution of 2 × 2 × 2 mm^3^, it is important to note that voxels that contain gray matter are still susceptible to partial volume effects if they fall on the boundary of gray and white matter, or gray matter and cerebrospinal fluid (CSF). We were able to appreciably diminish gray matter-CSF partial volume effects by using the free-water model, as well as including only values within three standard deviations of the mean.

To address the issue of partial volume between the white and gray matter boundary, we identified voxels in our gray matter ROIs where at least one neighboring voxel was labeled as white matter in the FreeSurfer parcellation, and removed these voxels from analysis. HFA was then recomputed for the remaining voxels, to verify that our primary results were not influenced by the presence of white matter at boundary voxels. Independent-two-samples *t*-tests were performed to determine whether there were differences in HFA between the two groups, controls and 22q11DS, in the brain regions that were found to differ significantly in our first analysis, namely the association and the paralimbic areas. After removing boundary voxels, the HFA in association areas (*M* = −0.0070, 95% CI [−0.011, −0.003], *t*(84) = −3.8, *p* = .0003, *d* = 0.83) was significantly higher in the 22q11DS group compared to the control group. HFA in the paralimbic areas (*M* = −0.008, 95% CI [−0.011, −0.005], *t*(84) = −5.2, *p* = .000001, *d* = 1.13) was also significantly higher in 22q11DS. We did not include a comparison of primary area, as this one was not statistically significant in the HFA comparisons between groups without boundary correction.

The *p*-values for these two regions remained statistically significant between groups following correction for partial volume effects of the gray-white matter boundary. Furthermore, the *p* and the *d* values for HFA, with boundary voxels removed, showed a similar trend to that seen in the analysis for HFA without boundary voxel removal. This corroborates that our main findings reflect increased variability in gray matter tissue structure in 22q11DS, excluding partial volume effect as a possible confound.

### 3.3 Heterogeneity in FA does not correlate with cortical thickness

We also investigated whether or not HFA was influenced by cortical thickness by computing Pearson's Product-Moment correlations to assess the relationship between HFA values and cortical thickness in each of the three functional areas using all subjects (controls and 22q11DS). There were no statistically significant correlations between these two variables for any of the areas (*r* < 0.15, *p* ≥ .05, *N* = 86). These results suggest that there is no association between cortical thickness and HFA, thereby mitigating the possibility that cortical thickness drove the changes observed in HFA between the groups.

### 3.4 Correlations between heterogeneity in FA and neuropsychological measures

**Fig. 4:**
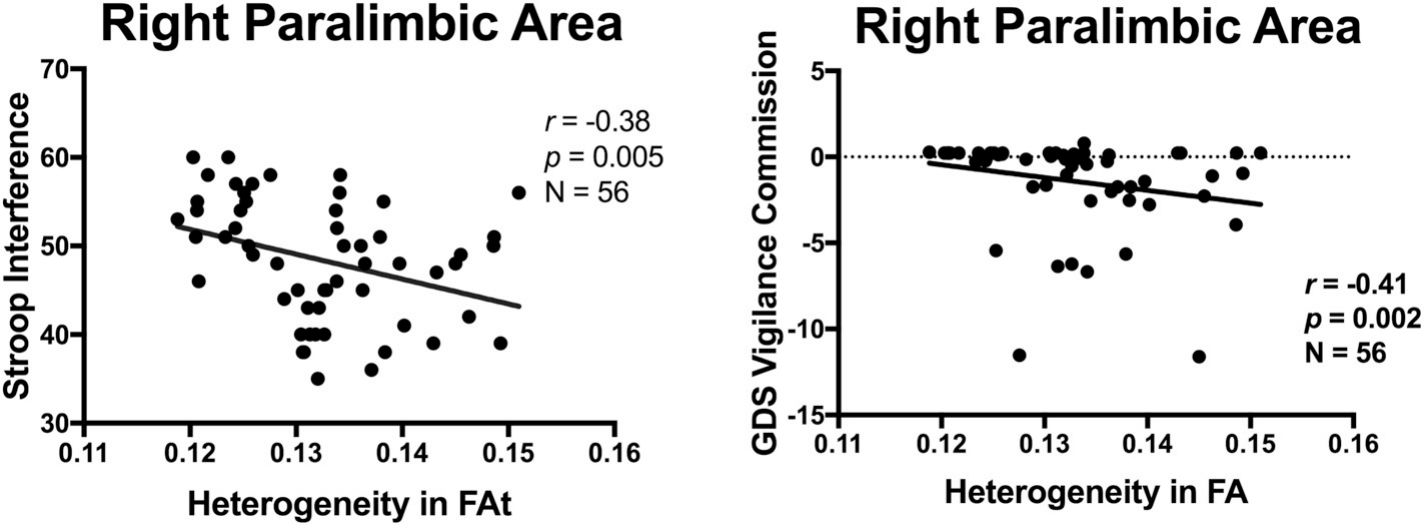
Association of heterogeneity in FA (HFA) and executive functioning in individuals with 22q11DS. Correlations between HFA in the right paralimbic area and two tests on response inhibition (Stroop Interference and Gordon Diagnostic System Vigilance Commission scores) are plotted.

We also explored the relationship between gray matter heterogeneity and subjects' cognitive functioning. For this purpose, we performed Spearman's Rho correlations between the neuropsychological scores and HFA values for each of the three brain regions (primary, association, paralimbic) separately for each group (healthy controls and 22q11DS). The two tests of response inhibition, the Stroop test (*rho* = −0.37, *p* = .005, *N* = 56) and the GDS Vigilance Commission test (*rho* = −0.41, *p* = .002, N = 56), correlated significantly with the HFA of the right paralimbic area in the 22q1DS group (Table 4, Fig. 4). These tests of response inhibition did not correlate significantly with HFA in any other areas (primary or association), or in the control group. None of the other *a priori* selected psychological tests (WCST, WAIS WMI, CVLT) correlated with the HFA for any area in the 22q11DS or in the healthy control group.

**Table 4:** Correlations between HFA and psychometric tests in individual with 22q11DS (N = 56).

		HFA primary area		HFA association area		HFA paralimbic area	
		Left	Right	Left	Right	Left	Right
**Stroop Interference T-score**	Spearman's rho	−0.230	−0.31	−0.263	−0.243	−0.278	**−0.372**^**⁎⁎**^
	p Signif. (2-tailed)	0.089	0.020	0.050	0.071	0.038	**0.005**
GDS Distractibility Commission *Z*-score	Spearman's rho	0.012	0.082	0.070	0.055	0.045	−0.292^⁎^
	p Signif. (2-tailed)	0.932	0.550	0.608	0.688	0.743	0.029
**GDS Vigilance Commission Z-score**	Spearman's rho	0.088	0.118	0.082	0.030	0.041	**−0.414**^**⁎⁎**^
	p Signif. (2-tailed)	0.520	0.386	0.547	0.825	0.766	**0.002**
Gordon CPT Vigilance Omission	Spearman's rho	−0.071	−0.099	−0.113	−0.084	−0.115	−0.168
	p Signif. (2-tailed)	0.603	0.468	0.406	0.540	0.401	0.217
WCST Perseverative Errors	Spearman's rho	−0.204	−0.094	−0.086	0.028	−0.045	0.012
	p Signif. (2-tailed)	0.132	0.489	0.528	0.840	0.744	0.933
WAIS WMI	Spearman's rho	0.024	−0.015	0.027	0.024	0.110	−0.006
	p Signif. (2-tailed)	0.862	0.913	0.844	0.858	0.422	0.962
California Verbal Learning Test List A	Spearman's rho	−0.027	−0.070	−0.062	−0.051	−0.051	0.174
	p Signif. (2-tailed)	0.843	0.611	0.651	0.709	0.711	0.199

HFA: Heterogeneity in Fractional Anisotropy; 22q: 22q11DS; ** Significant correlation at *p* < .01 (2-tailed)(in bold); * Significant correlation at *p* ≤ .05 (2-tailed).

GDS: Gordon Diagnostic System; CPT: Continous Performance Test; WCST: Wisconsin Card Sorting Test; WAIS WMI: Wechsler Adult Intelligence Scale Working Memory Index. Probability values *p* are given in bold face when significant after correction for multiple comparison.

## 4 Discussion

### 4.1 Variability in the microstructure of cortical gray matter in individuals with 22q11DS

The present study explored differences in gray matter microstructure between young adults with 22q11DS and healthy controls matched for age and gender. This is the first study to investigate alterations in gray matter microstructure in 22q11DS using dMRI, an imaging technique that has traditionally been applied to the analysis of white matter architecture *in vivo.* An advanced model of diffusivity was employed to compute gray matter fractional anisotropy (FA) values, which removes the signal arising from extracellular CSF areas, thereby reducing partial volume effects. Here, we analyzed average FA across different cortical areas categorized into three functional brain regions, namely primary, associative, and paralimbic areas. Furthermore, we employed an innovative measure, heterogeneity of FA (HFA), to explore the variability in gray matter tissue architecture in individuals with 22q11DS compared to healthy controls. Our analyses revealed that while FA did not differ between controls and 22q11DS subjects, heterogeneity in FA was significantly higher in the 22q11DS group in associative and paralimbic areas. No differences, however, were found between groups in primary areas.

The increase in HFA values observed in 22q11DS subjects suggest larger variance in the microstructural arrangement of cells and tissue in association and paralimbic areas in 22q11DS. The lack of a statistically significant difference in FA, on the other hand, suggests that on a regional level, the average anisotropy in diffusion is relatively the same in both groups. This highlights the importance of investigating several statistical variables in a region (including mean and variance) in order to fully understand the arrangement of gray matter tissue. While it is not known yet whether and what type of cellular changes are assessed by HFAt, one could speculate, based on the findings in the 22q11DS mouse model of displaced projection neurons and interneurons in the cortical layer 2/3 (Meechan et al., 2015), and also based on deficient migration of neural crest cells in early development in 22q11DS (Scambler, 2000), that changes in the cortex are present in individuals with 22q11DS and possibly revealed by the HFAt measurements.

Our findings of microstructural variability in the gray matter in association and paralimbic areas of the brain is in accordance with reported abnormalities in the microstructure (measured by FA) in white matter connecting these regions. Interestingly, white matter tracts that connect association areas, including the inferior longitudinal fasciculus (ILF), the middle longitudinal fasciculus (MdLF), the extreme capsule (EmC), and the inferior fronto-occipital fasciculus (IFOF), along with tracts connecting paralimbic regions, such as the cingulum, are reported to be altered in individuals with 22q11DS in our cohort (Olszewski et al., 2017; Kates et al., 2015; Kikinis et al., 2015; Tylee et al., 2017). Thus taken together, these findings suggest that related microstructural changes occur in both gray and white matter in young adults with 22q11DS.

### 4.2 Higher variability in cortical gray matter microstructure is associated with poorer response inhibition

We hypothesized that variability in the microstructure of gray matter would be associated with cognitive functioning in specific brain regions. Indeed, increased heterogeneity in the paralimbic areas was correlated negatively with scores on two separate tests of response inhibition, the Stroop and GDS Vigilance test, in subjects with 22q11DS (Table 4), but not in the control group. Our findings are thus in line with published findings in individuals with 22q11DS. First, and more specifically, deficits in response inhibition, using the Go/NoGo measure, a classical test of response inhibition, have been reported in children with 22q11DS (Shapiro et al., 2013). Second, subjects with 22q11DS performing the Go/NoGo test showed activation in the cingulate, a region of the paralimbic area, in a functional imaging study (Gothelf et al., 2007). However, the scores on other psychological tests assessing different domains of executive functioning and learning, including WCST, WAIS WMI and CVLT, did not correlate with HFA in association area. WCST is one of the most commonly used tests to assess executive functioning, and the WCST paradigm activates dorsolateral prefrontal cortex (DLPFC), a cortical region within the association area (Lie et al., 2006; Boschin et al., 2017; Konishi et al., 1999; Konishi et al., 1998). A likely explanation for the non-significant correlation between HFA and WCST may be that this functional brain region is too small in comparison to the entire association area, and therefore the effect may have been washed out. This could be addressed by subdividing the association area into smaller regions and rerunning the correlations tests in future studies.

To summarize, we demonstrated for the first time that reduced cognitive abilities are associated with increased variability in the microstructure of gray matter in individuals with 22q11DS.

### 4.3 Findings of microstructural abnormalities in gray matter in other neurodevelopmental disorders

Abnormalities in gray matter microstructure have also been reported in chronic and early course schizophrenia in neuroimaging studies (Harrison, 2000). The findings in schizophrenia are of interest to the current study because individuals with 22q11DS have of a high incidence of schizophrenia in adulthood (Schneider et al., 2014). One of the published study explored gray matter in individuals with chronic schizophrenia and bipolar disorder using a dMRI technique called Neurite Orientation Dispersion and Density Imaging (NODDI). The results of this study demonstrated abnormalities in gray matter microstructure in schizophrenia but not in bipolar disorder, suggesting that such abnormalities may be specific to schizophrenia (Zhang et al., 2012; Nazeri et al., 2016).

Extending findings of gray matter alterations to the early course schizophrenia, Seitz et al. (2017) demonstrated abnormalities in gray matter tissue structure in individuals with a first episode of schizophrenia. Abnormalities in gray matter heterogeneity in this population were reported in the frontal lobe, but not in the occipital or temporal lobe. In our study on individuals with 22q11DS, a population at high risk for developing schizophrenia in adulthood, we find that abnormalities in gray matter microstructure are present in association and paralimbic areas in young adulthood. This finding provides support for the hypothesis that abnormalities in gray matter microstructure seen in schizophrenia may be neurodevelopmental in origin, rather than indicative of degenerative processes, or associated with a psychotic state (Lewis and Levitt, 2002). With respect to 22q11DS, future longitudinal studies are needed to confirm whether or not increased heterogeneity of gray matter is characteristic of the genetic syndrome itself, or an aberration more specific to individuals who are at high risk for, and eventually go on to develop, psychosis.

Changes in the microstructure of gray matter have been further reported in individuals with other neurodevelopmental disorders, such as autism spectrum disorder (ASD) and attention deficit / hyperactivity disorder (ADHD), both of which are common in 22q11DS. Restriction spectrum imaging, a method to assess neurite density and neurite organization in gray matter, revealed reduction in neural density in parietal regions in individuals with ASD when compared to healthy subjects (Carper et al., 2016). Similarly, in subjects with ADHD, preliminary findings of microstructural changes in the gray matter in the prefrontal cortex have been reported using diffusional kurtosis imaging (Helpern et al., 2011). While only very few studies have investigated the microstructure in gray matter *in vivo* to-date, these studies and emerging new methods, hold promise for mapping abnormalities of the cortex, which are present in disorders with neurodevelopmental delays.

### 4.4 Study limitations

Studies performed on individuals with a rare genetic disorder are usually limited by the small number of subjects, as well as by inclusion of individuals that span an age range in which dMRI metrics may not be linear. The 22q11DS syndrome, with an incidence of 1:4000 in the general population, is a rare genetic syndrome. Nonetheless, our study sample is of a narrow age range (18–26 years old) and sufficiently large - 56 young adults with 22q11DS and 30 control subjects - to enable several findings with large effect sizes. Nonetheless, we cannot rule out the fact that other non-significant findings may be negatively impacted by low statistical power. Another potential limitation is the effect of psychotropic medication and the use of recreational drugs on microstructural alterations in gray matter. However, only a small number of 22q11DS were receiving psychotropic medications in this study (6 out of 56), and for this reason we do not think our findings are confounded by medications. Further, the prevalence of recreational drug use was similar between the control group and the 22q11DS group.

### 4.5 Conclusion and implications for future research

In summary, we report abnormalities in gray matter microstructure in young adults with 22q11DS. Changes in microstructure were limited to brain areas involved in higher level cognitive processing, such as association and paralimbic areas in the brain. Microstructure of gray matter was assessed using dMRI and the output measure was heterogeneity in FA (HFA). Furthermore, HFA correlated negatively with tests of response inhibition, suggesting that increased heterogeneity in gray matter is associated with weaker impulse control abilities. Future research should investigate whether HFA is increased in other neurodevelopmental disorders and how this increase may be related to neurocognition and psychopathology.

## Compliance with ethical standards

The authors declare that they have no conflict of interest. All procedures performed in studies involving human participants were in accordance with the ethical standards of the institutional and/or national research committee and with the 1964 Helsinki declaration and its later amendments or comparable ethical standards. All participants signed an Informed Consent form, and the study was approved by the institutional review board at SUNY Upstate Medical University, Syracuse, NY and by the Partners Human Research Commission, Boston, MA.

## Funding resources

This work was supported by funding from the National Institutes of Health grants MH064824 to WRK, MH106793 to ZK, R01MH097979 to YR, AG042512 and AT008865, R01AG042512, R21AT008865, R21DA042271, R01MH111917 to NM, R01HD090641 to SB, R01MH108574, R01MH085953 to OP VA Merit Award I01 CX000176-06 to MES.
